# Facet‐Control versus Co‐Catalyst‐Control in Photocatalytic H_2_ Evolution from Anatase TiO_2_ Nanocrystals

**DOI:** 10.1002/open.202200010

**Published:** 2022-02-03

**Authors:** Shanshan Qin, Lancang Shui, Benedict Osuagwu, Nikita Denisov, Alexander B. Tesler, Patrik Schmuki

**Affiliations:** ^1^ Department of Materials Science and Engineering WW4-LKO University of Erlangen-Nuremberg Martensstrasse 7 91058 Erlangen Germany; ^2^ Chemistry Department King Abdulaziz University 80203 Jeddah Saudi Arabia Kingdom; ^3^ Regional Centre of Advanced Technologies and Materials Palacky University Listopadu 50 A 772 07 Olomouc Czech Republic

**Keywords:** anatase TiO_2_, crystal facet engineering, Pt co-catalyst, photocatalytic H_2_ evolution

## Abstract

Titanium dioxide (TiO_2_) and, in particular, its anatase polymorph, is widely studied for photocatalytic H_2_ production. In the present work, we examine the importance of reactive facets of anatase crystallites on the photocatalytic H_2_ evolution from aqueous methanol solutions. For this, we synthesized anatase TiO_2_ nanocrystals with a large amount of either {001} facets, that is, nanosheets, or {101} facets, that is, octahedral nanocubes, and examined their photocatalytic H_2_ evolution and then repeated this procedure with samples where Pt co‐catalyst is present on all facets. Octahedral nanocubes with abundant {101} facets produce >4 times more H_2_ than nanosheets enriched in {001} facets if the reaction is carried out under co‐catalyst‐free conditions. For samples that carry Pt co‐catalyst on both {001} and {101} facets, faceting loses entirely its significance. This demonstrates that the beneficial role of faceting, namely the introduction of {101} facets that act as electron transfer mediator is relevant only for co‐catalyst‐free TiO_2_ surfaces.

## Graphical abstract

**The role of reactive facets of anatase** TiO_2_ crystallites on photocatalytic H_2_ evolution is examined in this work. Pt co‐catalyst‐loaded and Pt‐free preferentially faceted nanocrystals are compared. As expected, both types of Pt‐decorated TiO_2_ nanocrystals demonstrate significantly enhanced photocatalysis yet comparable H_2_ evolution indicating that the beneficial effect of faceting is lost for the Pt‐decorated nanocrystals.

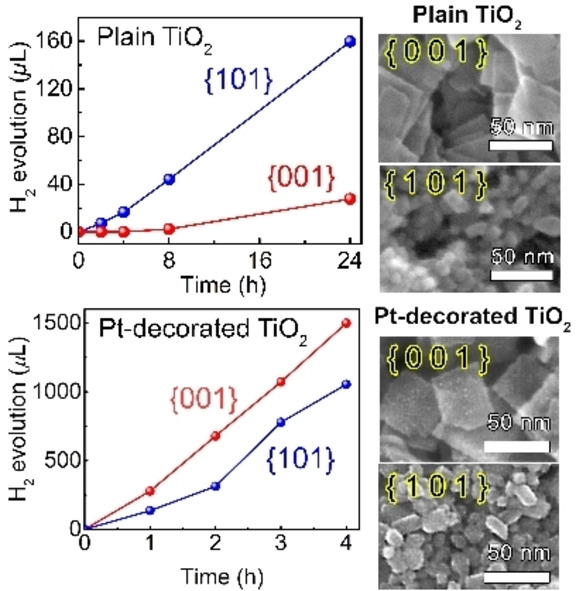

## Introduction

With the depletion of fossil fuel and growing demand for green sustainable energy sources, photocatalytic H_2_ evolution attracts increasing attention in recent decades.^[1]^ Since the pioneering work of Fujishima and Honda on photocatalytic water splitting,^[2]^ photocatalytic H_2_ evolution using semiconductor materials has become a most promising clean energy technology with minimal impact on the environment.^[3]^ Photocatalytic H_2_ generation is based on the interaction of light with a suitable semiconductor and a reaction of the generated charge carriers with an aqueous environment (with or without sacrificial agents) to form the desired H_2_. Essential reaction steps involve (*i*) absorption of photons and generation of electron (*e* ^−^) – hole (*h* ^+^) pairs, (*ii*) charge carrier separation, *(iii)* migration of *e* ^−^ or *h* ^+^ from the bulk to the surface, and, finally, (*iv*) charge carrier reaction (reduction of H^+^ to H_2_ and a complementary photo‐oxidation reaction) at the surface of a photocatalyst. The overall performance of a photocatalyst is strongly determined by the kinetics of these four steps.

The semiconducting material absorbs photons with energies higher than its bandgap.^[4]^ Numerous photocatalytic materials have been investigated but among available developed materials, titanium dioxide (TiO_2_) is still considered one of the most promising photocatalysts due to its strong oxidizing and reducing capabilities, long‐term stability, non‐toxic nature, abundance, and low cost.^[5]^ TiO_2_ fulfills the thermodynamic conditions for full water splitting, that is: the conduction band (CB) edge is lower than the H^+^/H_2_ reduction potential (0 V vs. NHE), while the valence band (VB) edge is higher than the H_2_O/O_2_ oxidation potential (+1.23 V vs. NHE).^[6]^ Nevertheless, the overall performance of the photocatalyst is often determined by kinetic factors^[7]^ and here namely the charge transfer reactions that depend on a variety of material parameters.

TiO_2_ nanostructures exist mainly in three crystallographic polymorphs, i. e., anatase, rutile, and brookite.^[8]^ Due to the longest lifetime of photocarriers, anatase is commonly the “best‐performing” semiconducting form of TiO_2_, which makes it the most popular polymorph in the fields of catalysis, photocatalysis, and dye‐sensitized solar cells. It was shown that the photocatalytic water splitting performance of TiO_2_ crystals varies with the surface physicochemical properties such as size, shape, crystal structure, and amount of exposed facets.^[7b]^ Recently, great progress has been achieved in crystal facet engineering of anatase TiO_2_ nanocrystals.^[9]^ For instance, the reduction and oxidation reactivity of different anatase crystal facets were investigated by Majima et al. through single‐particle chemiluminescence imaging.^[10]^ It was found that the H_2_ evolution reaction of different anatase TiO_2_ crystal facets can be rated as {101}>{001}>{100}, while that of O_2_ evolution reaction is {101}≈{001}≈{100}.

Accordingly, the development of TiO_2_ crystals with specific facets has attracted wide interest. The anatase (101) plane has the lowest surface energy being the most stable surface.^[11]^ Yang et al. synthesized anatase TiO_2_ crystals with 47 % {001} and 53 % {101} facets,^[12]^ which was a breakthrough in the synthesis of high‐quality anatase TiO_2_ crystals with a high percentage of reactive high energy {001} facets. In the meantime, different strategies to control the synthesized facets, such as wet‐chemistry (hydrothermal, solvothermal, and nonhydrolytic), gas oxidation, topotactic transformation, crystallization from amorphous TiO_2_, and epitaxial growth have been developed.^[11]^ Among them, the hydrothermal method is the most common technique for tailoring the exposed crystal facets due to the possibility of manipulating the nucleation and growth processes by controlling the experimental conditions. For instance, introducing capping agents (e. g., appropriate organic molecules, inorganic ions, or their mixtures) in the hydrothermal synthesis allows decreasing the surface energies of different surfaces, which has been extensively used to control the growth rates along different orientations.[^12^, ^13^] Consequently, nanocrystals with tunable percentages of particular facets can be formed.

In the present work, we synthesize anatase TiO_2_ nanocrystals with a predominant amount of either {001} or {101} facets by hydrothermal methods leading to the formation of TiO_2_ nanosheets (NS) with {001} facets or TiO_2_ octahedral nanocubes (Oct) with {101} facets.[^9^, ^12^] The consensus exists in the literature that faceting is always beneficial and, in particular, an advantageous photocatalytic performance of {101}‐faceted anatase TiO_2_ nanocrystals was provided against all other facets.[^10^, ^14^] Here we show, however, that this is only appropriate for the co‐catalyst‐free state of TiO_2_ nanocrystals. When Pt is deposited on the entire nanocrystal surface, unfavorable {001}‐faceted TiO_2_ nanosheets demonstrated 400‐fold enhancement in their photocatalytic H_2_ evolution performance as compared to their plain counterparts and even 2‐fold enhancement as compared to favorable {101}‐faceted Pt‐decorated anatase TiO_2_ octahedral nanocrystals. This was achieved by the decoration of the entire TiO_2_ nanocrystals surface, that is, both {001} and {101} facets, using immersion deposition process rather than the selective decoration of {101} facets as was demonstrated in the literature achieved by the commonly used photodeposition approach.^[15]^

## Results and Discussion

**Scheme 1:**
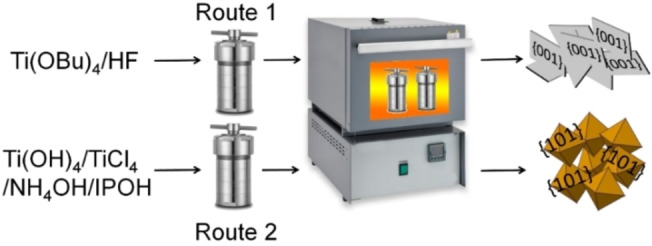
Schematic representation of the hydrothermal processes forming anatase TiO_2_ NS (route 1) or Oct (route 2), enriched by either {001} or {101} facets, respectively.

**Figure 1:**
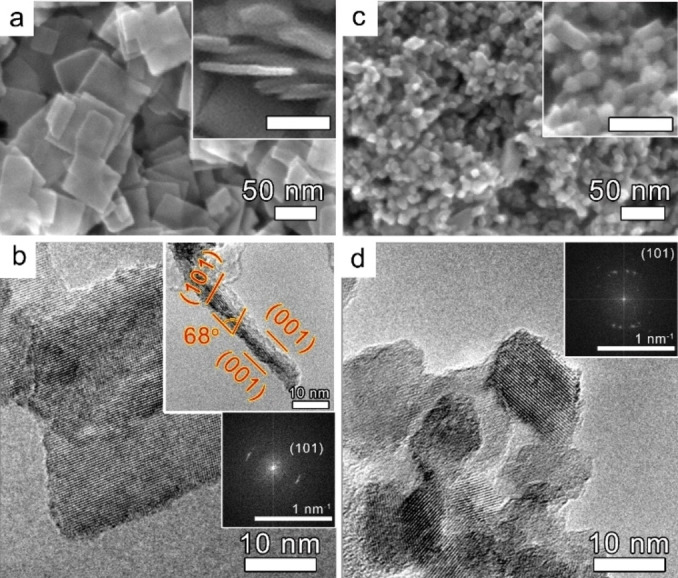
Representative FE‐SEM and HR‐TEM images of hydrothermally synthesized anatase TiO_2_ (**a**–**b**) NS and (**c**–**d**) Oct. Insets in (b, d) show a fast Fourier transform of the corresponding TEM images. The inset image in (b) is the top‐view of the TiO_2_ NS.

Scheme 1a demonstrates the hydrothermal approaches used in this work to synthesize anatase TiO_2_ nanosheets (NS) and octahedral nanocubes (Oct) enriched by either {001} or {101} facets, as described in the experimental section and as given in Refs. 9 and 11. Figure 1a–b shows representative FE‐SEM and HR‐TEM images of the TiO_2_ NS revealing a well‐defined faceted morphology. The synthesized anatase TiO_2_ NS are 43.5±19.3 nm wide in the basal plane and 5.9±1.3 nm thick (Figure 1a–b). TEM images of NS demonstrate lattice fringes with 0.35 nm spacing characteristic for anatase {101} orientation (*d_101_* =0.352 nm, JCPDS card #21‐1272). Notably, the determined {101} crystalline planes in NS show a 68° angle to the lateral view of the basal facets, in agreement with the theoretical angle between {101} and {001} facets,^[16]^ therefore, the latter can be assigned to {001} surfaces.

The amount of {001} facets can be evaluated according to the following equation [Eq. 1]:

(1) $$P_{\left\{001\right\}}=\frac{2l^{2}}{2l^{2}+4ld}\times 100\;\%$$

where *P*_{001}_ indicates the amount of {001} facets, *l* and *d* are the mean length and thickness of TiO_2_ NS, respectively.^[17]^ According to the calculations, the amount of {001} facets in the synthesized TiO_2_ NS is 87.9 %. Brunauer‐Emmett‐Teller (BET) evaluation of NS yields a specific surface area of the obtained TiO_2_ NS as 86.1 m^2^ g^−1^.

Figure 1c–d shows representative FE‐SEM and HR‐TEM images of the anatase TiO_2_ octahedral nanocrystals (Oct). It was previously demonstrated that a low supersaturation benefits the growth of {101} facets, that is, reducing the amount of Ti(OH)_4_ precursor favors the formation of octahedral TiO_2_ nanocrystals.^[11]^ Therefore, the hydrothermal process of the formation of anatase TiO_2_ Oct was optimized along these lines (Table S1 and Figure S1, Supporting Information). Figure S1 demonstrates the influence of the amount of Ti(OH)_4_ precursor and hydrothermal process temperature on the obtained TiO_2_ Oct. As shown, fixing the amount of precursor to 1.0 g produces the most homogeneous TiO_2_ crystals (Figure S1g). The rise in the process temperature contributes to their larger size. Furthermore, rapid quenching of the growth period is beneficial to a prominent crystallization and size distribution.^[18]^ Therefore, the hydrothermal reaction of anatase TiO_2_ Oct was quenched rapidly by cooling down the autoclave using cold tap water. Nevertheless, when the reaction duration reaches a critical point, a longer reaction time does not contribute significantly to the formation of larger TiO_2_ Oct, which can be attributed to a fixed amount of reactant; still, a more uniform size distribution of TiO_2_ Oct was obtained (Figure S1e–f). According to our experiments, the optimized amount of Ti(OH)_4_ precursor (1.0 g), hydrothermal temperature (230 °C), and reaction duration of 40 h, as well as rapid reaction quenching, are crucial to obtain the most uniform octahedral TiO_2_ Oct with a large percentage of {101} facets. The size distribution of anatase octahedral TiO_2_ obtained under optimal conditions was estimated from FE‐SEM images and found as 12.1±4.2 nm. Considering the particle size and uniformity, the amount of {101} facets can be estimated according to Equations (2) and 3:

(2) $$h=\sqrt{{\left(\frac{b-a}{2}\right)}^{2}+{\left(\frac{l}{2}\right)}^{2}}$$

(3) $$P_{\left\{101\right\}\;facets}=\frac{8\times \frac{(a+b)\times h}{2}}{8\times \frac{(a+b)\times h}{2}+2\times a^{2}}\times 100\;\%$$

where *a* and *b* refer to the length of the two parallel sides of an isosceles trapezoid, *l* is the height of crystal, *h* is the height of the trapezoidal facet, and *P*_{101}_ *facets* stand for the percentage of {101} facets.^[9]^ In this study, the *a*, *b*, *l*, and *h* parameters for the optimized sample were estimated as 2.5, 12.5, 15.0, 9.0 nm, respectively, resulting in the 97.7 % fraction of {101} facets. The corresponding BET isotherm demonstrates that the specific surface area of anatase TiO_2_ Oct is 111.7 m^2^ g^−1^, that is, 1.3 times larger than that of NS.

**Figure 2:**
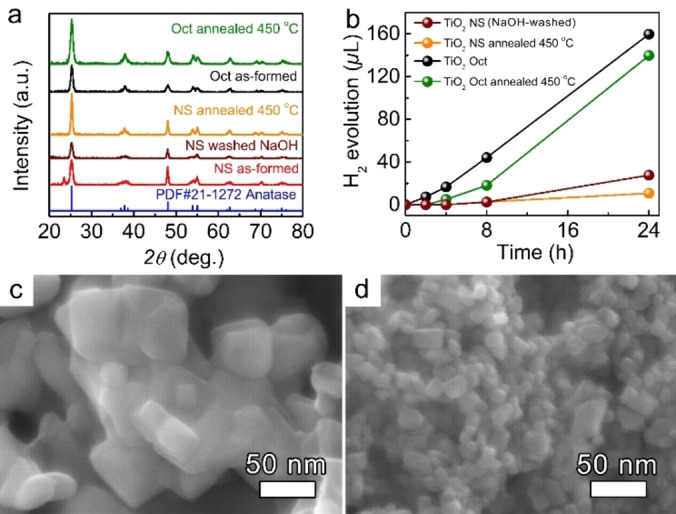
(**a**) XRD patterns of the as‐formed, NaOH‐washed, and annealed at 450 °C TiO_2_ NS, and as‐formed and annealed TiO_2_ Oct. (**b**) 24 h photocatalytic H_2_ evolution was obtained from bare and annealed anatase TiO_2_ NS, and as‐formed and annealed Oct in 50 : 50 vol.% H_2_O:MeOH electrolyte. (**c**–**d**) HR‐SEM images of TiO_2_ NSs (c) and Oct (d) after annealing at 450 °C for 1 h.

The crystallinity of the as‐formed NS and Oct was analyzed by X‐ray diffraction (XRD) (Figure 2a). In both cases, the XRD patterns show sharp characteristic peaks of anatase TiO_2_ at *2θ*=25.3°, 37.8°, 38.6°, 48.1°, 54.0°, 55.1°, 62.7°, 70.5°, and 75.1° corresponding to (101), (001), (112), (200), (105), (211), (220), and (215) crystallographic plains, respectively (JCPDS card #21‐1272).^[20]^ However, the TiO_2_ NS photocatalyst shows an additional peak at *2θ*=23.61° that can be attributed to TiOF_2_ (JCPDS card #08‐0060) formed during the synthesis in HF, used as a capping reagent, while octahedral nanocrystals were synthesized under the F‐free conditions, that is, Ti(OH)_4_ was utilized as the Ti precursor and isopropanol as capping agent and solvent. Therefore, prior to comparing the photocatalytic performance of anatase TiO_2_ nanocrystals with various faceting, the TiOF_2_ phase was removed.

We used two approaches to obtain pure anatase TiO_2_ NSs: (*i*) thermal treatment of as‐formed NSs in air, and (*ii*) washing of the as‐formed NSs in an aqueous NaOH solution. After the thermal treatment at 450 °C for 1 h, the TiOF_2_ peak in NSs disappears (Figure 2a, orange pattern). The corresponding annealed NS photocatalyst shows an increased peak intensity at *2θ*=25.3° and 37.8°, indicating an improvement in the crystallinity. However, TiO_2_ NS enriched in {001} facets deform (sinter) and agglomerate after the annealing as evident from Brunauer‐Emmett‐Teller (BET) measurements. The thermal treatment process leads to a drop in their surface area from 86.1 to 30.3 m^2^ g^−1^ (Figure 2c, and Table 1). The agglomeration and further sintering can be explained by the loss of the F‐terminated layer present mainly on {001} facets eventually leading to an increase in their surface energy.[^11^, ^19^] In contrast, the TiO_2_ NSs after washing in an aqueous solution of 0.1 M NaOH show a total disappearance of the TiOF_2_ peak while still preserving their NS morphology and anatase TiO_2_ crystalline structure (Figure 2a, dark red pattern, and Figure S2a–b). The EDX analysis confirms the diminishing of fluorine in the NaOH‐washed TiO_2_ NSs as compared to their as‐formed counterparts (Figure S2c–d). Therefore, we used the NaOH‐washed anatase TiO_2_ NS nanocrystals enriched in {001} facets to analyze their photocatalytic performance and compare it to the anatase TiO_2_ octahedral nanocrystals enriched in {101} facets. Noteworthy, in contrast to deformation and agglomeration of the NS, an intact octahedral morphology of the anatase TiO_2_ nanocrystals was observed upon annealing in air at 450 °C for 1 h (Figure 2d). This is in line with the literature since the anatase (101) plane has the lowest surface energy making it the most stable surface.^[11]^

**Table 1:** BET measurements of the as‐formed and annealed at 450 °C for 1 h in air NS and Oct nanocrystals.

Samples	BET [m^2^ g^−1^]
NS as‐formed	86.1
NS annealed at 450 °C	30.3
Oct as‐formed	111.7
Oct annealed at 450 °C	112.5

Since the surface composition can affect the H_2_ production performance, X‐ray photoelectron spectroscopy (XPS) analysis of the as‐formed TiO_2_ Oct and NaOH‐washed TiO_2_ NSs was performed, and the results are summarized in Figure S3. In both cases, the high‐resolution Ti 2p XPS spectrum consists of two peaks at the binding energies of 458.3 eV and 464.1 eV (Figure S3a–b), corresponding to the Ti^4+^2p_3/2_ and 2p_1/2_ oxidation state of Ti, respectively (Figure S3a–b).^[20]^ In the case of the O 1s spectra, the main peak of NaOH‐washed TiO_2_ NSs is centered at 529.3 eV, and a shoulder at 530.5 eV is visible. The deconvolution of the O 1s peak leads to two main components: at 529.3 eV (84 %) corresponding to the bulk oxygen in TiO_2_ (Ti−O−Ti linkages), and 530.5 eV (14 %), corresponding to surface hydroxyl groups (Ti−OH) (Figure S3c).^[20]^ At the same time, the as‐formed TiO_2_ Oct demonstrates a longer shoulder that was deconvoluted into three components: 529.3 eV (82 %), 530.6 eV (9 %), and 531.70 eV (6 %) corresponding to Ti−O−Ti, Ti−OH, and C−O/C=O bonds, respectively, as well as 533.0 eV (3 %), corresponding to H_2_O (Figure S3d).[^20^, ^21^] Here the signal from C−O/C=O bonds and H_2_O can be attributed to organic species and water adsorbed from the atmosphere. As shown, the XPS measurements demonstrate the comparable surface composition in both samples.

Figure 2b demonstrates the photocatalytic activity of both anatase TiO_2_ NS and Oct, as well as the photocatalytic performance of their corresponding annealed counterparts, represented by the amount of H_2_ generated in the 50 : 50 vol.% H_2_O:MeOH mixture. It should be noted that TiO_2_ NSs, used in all further experiments, are NaOH‐washed, for simplicity these samples will be named “bare” TiO_2_ NSs. The TiO_2_ NS or Oct photocatalysts were illuminated by UV light at *λ*=365 nm for 24 h and H_2_ evolution was measured by gas chromatography. As shown, the H_2_ evolution performance for both bare and annealed NS is significantly lower than that of as‐formed and annealed Oct being in good agreement with the literature. The H_2_ evolution rate was calculated and found as 2.30×10^2^ and 13.25×10^2^ *μ*L g^−1^ h^−1^ for bare NS and as‐formed Oct, respectively, demonstrating the >4‐fold enhanced photocatalytic activity (normalized to the available surface area according to the BET measurements) of Oct as compared to their NS analogs.

As was extensively discussed in literature, reaction sites for effective reduction reaction are located preferentially on the {101} facets – they act as electron exit sites of the anatase TiO_2_ crystal, whereas {001} facets act as hole exit sites.[^10b^, ^22^] Our results of H_2_ evolution as shown in Figure 2b are well in line with the literature proofing again that the effective reduction, that is, H_2_ evolution, is preferentially located on the {101} facets of the TiO_2_ crystal rather than the {001} facets emphasizing once more a beneficial contribution of the exposed crystal facets on the photocatalyst efficiency.[^10b^, ^11^, ^22^] In our case, TiO_2_ octahedral nanocrystals with their large amount of {101} facets provide facilitated electron transfer towards H_2_ evolution reaction as compared to TiO_2_ NS, enriched mainly by {001} facets. Besides, a slight photocatalytic enhancement of TiO_2_ Oct can be further ascribed to their 1.3 times larger surface area.

**Figure 3:**
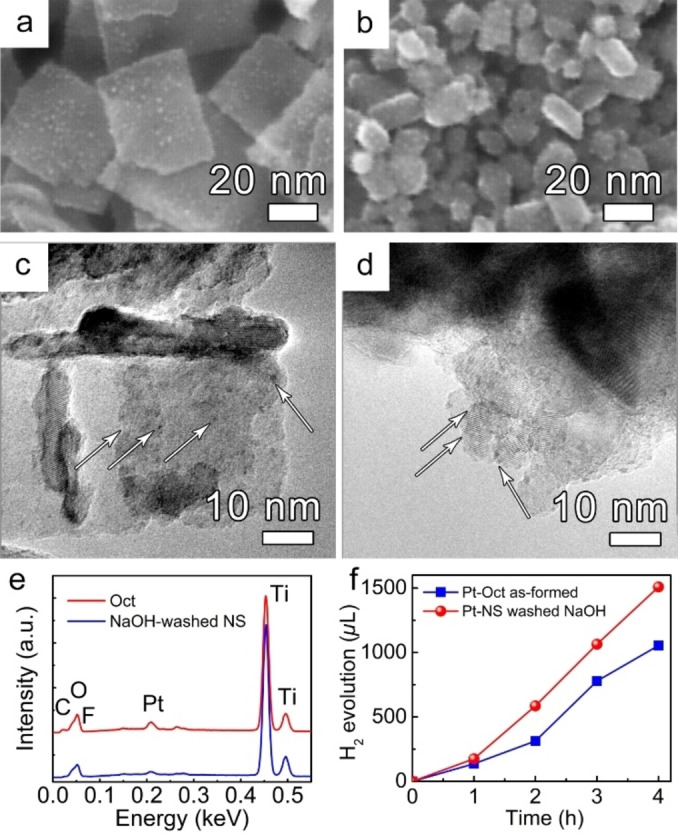
(**a**–**b**) SEM and (**c**–**d**) TEM images and (**e**) corresponding EDX spectra of Pt immersion‐deposited anatase TiO_2_ (**a**, **c**) bare NS and (**b**, **d**) as‐formed Oct. (**f**) Photocatalytic H_2_ evolution was obtained from anatase bare TiO_2_ NS−Pt and as‐formed Oct−Pt in 50 : 50 vol.% H_2_O:MeOH electrolyte.

Although, the faceting of the crystals is successful in enhancing charge carrier transfer, in most practical applications, photocatalytic reactions targeting a maximum of H_2_ evolution from anatase precursors widely employ co‐catalysts such as Pt, Au, Ru, or Pd.^[23]^ To examine whether faceting is still beneficial after decorating the TiO_2_ nanocrystals by a noble metal, Pt nanoclusters were deposited on the bare NS and as‐formed Oct using the immersion‐deposition technique as outlined in the experimental section. In contrast to photo‐deposition, the immersion‐deposition produces uniform decoration of Pt nanoclusters. The as‐formed Pt‐decorated TiO_2_ NS and Oct samples were studied by HR‐SEM and TEM analysis. Figure 3a–e show the SEM, TEM images, and SEM‐EDX elemental analysis of TiO_2_ NS and Oct after the Pt immersion‐deposition process. The presence of Pt nanoclusters can be observed by the appearance of bright dots on the SEM images (Figure 3a–b). As shown, Pt clusters cover the entire TiO_2_ NS and Oct surface, that is, Pt nanoclusters are equally deposited on both {101} and {001} facets. The dark Pt dots on the TEM images indicate the formation of a cluster size of Pt in the range of 0.95±0.22 nm (Figure 3c–d). SEM‐EDX elemental analysis of TiO_2_ NS and Oct after the Pt immersion‐deposition confirms a comparable amount of Pt loading for the two morphologies (Figure 3e).

Figure 3f demonstrates the photocatalytic H_2_ evolution of both the Pt‐decorated TiO_2_ bare NS and as‐formed Oct samples in the 50 : 50 vol.% H_2_O:MeOH mixture. The samples were illuminated by UV light at *λ*=365 nm for 4 h and H_2_ evolution was measured every 1 h. As expected, Pt decoration leads to the significant enhancement in the H_2_ evolution rate for both facetted TiO_2_ nanostructures, while in both these cases, the photocatalytic H_2_ evolution rates demonstrate the same order of magnitude. The H_2_ evolution rates of Pt‐decorated samples were estimated as 74.43×10^3^ and 52.71×10^3^ *μ*L g^−1^ h^−1^ for the Pt‐decorated bare NS and as‐formed Oct, respectively. Furthermore, Pt‐decorated bare anatase TiO_2_ NS enriched in {001} facets demonstrate ∼2‐fold enhanced photocatalytic performance over Pt‐decorated as‐formed TiO_2_ Oct enriched in {101} facets (normalized to the entire surface area of NS and Oct). The latter emphasizes that the {101} preferred faceting matters strongly in the case of plain anatase TiO_2_ nanostructures, that is, the preferential electron transfer is facet‐determined (rate‐determining step in heterogeneous reactions). Once decorated by Pt co‐catalyst, the Schottky junction is established leading to facile charge‐carrier transportation and separation reducing the recombination rate as well as providing efficient H^0^ recombination centers, that is, electric effects of Pt deposition becomes overall rate‐controlling; thus, the advantage of faceting that is a faster electron transfer kinetics on {101} facets vanishes entirely.

## Conclusion

Anatase TiO_2_ nanosheets enriched by {001} facets as well as anatase TiO_2_ octahedral nanostructures enriched in {101} facets were synthesized and compared regarding their photocatalytic H_2_ evolution performance with and without Pt photocatalysis. The plain TiO_2_ nanocrystals of both types were decorated by immersion‐deposited Pt clusters. In contrast to photo‐deposition, immersion‐deposited Pt clusters of sub‐nm‐size were obtained on the entire TiO_2_ nanocrystal surface, that is, on both {001} and {101} facets. Unlike octahedral nanostructures that improved their H_2_ evolution rate by one order of magnitude (∼40‐fold enhancement), TiO_2_ nanosheets demonstrated >400‐fold enhancement in their H_2_ evolution rate once decorated by Pt clusters. This study indicates clearly that the preferential electron transfer is facet‐determined, that is, rate‐determining step in heterogeneous reactions, for co‐catalyst‐free anatase TiO_2_ only. Once Pt‐decorated, both {001} and {101}‐enriched anatase TiO_2_ nanocrystals demonstrate comparable H_2_ evolution rates.

## Experimental Section

*Materials*: Ti(OBu)_4_ (98 %, Sigma‐Aldrich), HF (47 %, Carl Roth), TiCl_4_ (99.9 %, Acros Organics), H_2_PtCl_6_ ⋅ 6H_2_O (40.17 % metallic Pt weight concentration, Metakem), HCl (37 %, Sigma‐Aldrich), isopropanol (99.5 %, Sigma‐Aldrich), and NH_3_ ⋅ H_2_O (25 wt %, Merck) were used as received without further purification.

*Synthesis of anatase TiO_2_ nanosheets (NS) enriched by {001} facets*: In a typical synthesis procedure, 1.2 mL HF was added dropwise to 10 mL of Ti(OBu)_4_ placed in a 250 mL Teflon liner, under stirring at room temperature. The stirring was continued for the next 40 min before the Teflon containing the mixture was sealed in an autoclave, which was eventually transferred to a preheated oven at 200 °C. The reaction was completed after 24 h and the autoclave was allowed to cool in the oven naturally. After the hydrothermal reaction, the precipitates were collected and washed 3 times with ethanol and distilled water each, then dried in the oven at 75 °C overnight. To remove TiOF_2_ crystalline phase, the as‐formed NSs were rather annealed in air at 450 °C for 1 h or washed in NaOH solution. The latter was performed by dispersing the as‐formed TiO_2_ NSs in an aqueous solution of 0.1 M NaOH for 24 h under vigorous stirring followed by centrifugation at 4000 rpm and drying in the oven at 75 °C overnight.

*Synthesis of octahedral anatase TiO_2_ nanocubes (Oct) enriched by {101} facets*: Initially, Ti(OH)_4_ precursor was synthesized by adding 6.6 mL TiCl_4_ to aqueous HCl dropwise under vigorous stirring in an ice bath. The TiCl_4_ solution was then added to 5.5 wt.% NH_4_OH dropwise under stirring. 4 wt.% NH_4_OH was used to adjust the pH value to 7.0. After aging at room temperature for 2 h, the suspension was centrifuged and the precipitate was washed twice with DI water and ethanol. 1.0 g of Ti(OH)_4_ precursor was dispersed in the mixture of 15 mL DI water and 15 mL isopropanol. After both 30 min stirring and ultrasonication, it was transferred to a 100 mL Teflon autoclave for hydrothermal synthesis in the oven (230 °C, 40 h). Upon cooling down to room temperature, the white powder could be separated by centrifugation at 4000 rpm for 10 min, followed by washing with deionized water and ethanol, each step was repeated 3 times. A series of Ti(OH)_4_ precursors were synthesized by changing the amount of HCl and NH_4_OH as listed in Table S1. A series of TiO_2_ Oct were synthesized by changing the amount of precursor, reaction temperature and time, and the cooling method as listed in Table S2. The obtained as‐formed Oct was annealed in air at 450 °C for 1 h.

*Pt immersion‐deposition process of as‐formed TiO_2_ NS and Oct*: 100 mg of hexachloroplatinic(IV) acid hydrate (H_2_PtCl_6_ ⋅ 6H_2_O) was dissolved in 100 mL deionized water under vigorous stirring. After stirring for 10 min, 150 mg of either TiO_2_ as‐formed NS or as‐formed Oct were added to this solution and the slurry was sonicated ultrasonically for 1 h. Upon completion, the nanoparticles were washed by centrifuging and rinsing with deionized water three times. Finally, the washed powders were collected and dried in air at 70 °C for 12 h.

*Characterization methods*: A field‐emission scanning electron microscope (FE‐SEM, Hitachi, S4800) equipped with an energy dispersive X‐ray detector (EDAX Genesis, fitted to SEM chamber) was used for characterization of the morphology and chemical composition of the samples. The crystallographic orientation of the surface exposed crystal facets for the prepared samples was verified using the high‐resolution transmission electron microscopy (HR‐TEM, Philips CM30). The crystal phase structure of the samples was obtained by X‐ray diffraction (XRD, X'pert Philips PMD diffractometer) operating with graphite monochromatized Cu_Kα_ radiation (wavelength: *λ*=1.54056 Å). The composition and the chemical state of the TiO_2_ nanostructures were characterized using XPS (PHI 5600, US). The spectra were shifted according to the C 1s signal at 284.8 eV, and the peaks were fitted by MultiPak software. The specific surface area was performed through N_2_ adsorption/desorption measurements at 77 K on a volumetric gas adsorption analyzer (Autosorb iQ XR, Anton‐Paar Quanta Tec, USA) up to 0.965. Before the analysis, the sample was degassed under high vacuum (10^−7^ Pa) at 200 °C for 12 h, while high purity (99.999 %) N_2_ and He gases were used for the measurements. The Brunauer‐Emmett‐Teller surface area (BET) was determined concerning Rouquerol criteria for N_2_ isotherm. A gas chromatograph (GC: GCMS‐QO2010SE, Shimadzu) with a thermal conductivity detector was used to measure the amount of H_2_ generated. The sample was continuously stirred during irradiation and GC measurements were conducted to evaluate the amount of generated H_2_.

*Photocatalytic H_2_ evolution measurements*: In a typical experiment, 5 mg TiO_2_ NS or Oct were dispersed in the 10 ml mixture of water and methanol (=50 : 50 vol.%) solution in a quartz tube. After Ar purging for 20 min and ultrasonic treatment for 15 min, this system was UV irradiated (*λ*=365 nm, 65 mW cm^−2^) under stirring conditions. The amount of generated H_2_ was detected by the gas chromatography‐mass spectrometry system (GCMS‐QP2010 SE).

## Conflict of interest

The authors declare no conflict of interest.

1

## Supporting information

As a service to our authors and readers, this journal provides supporting information supplied by the authors. Such materials are peer reviewed and may be re‐organized for online delivery, but are not copy‐edited or typeset. Technical support issues arising from supporting information (other than missing files) should be addressed to the authors.

Supporting Information

## Data Availability

The data that support the findings of this study are available from the corresponding author upon reasonable request.
