# Supplementary material for: Facet‐Control versus Co‐Catalyst‐Control in Photocatalytic H2 Evolution from Anatase TiO2 Nanocrystals
Source: ChemistryOpen. 2022 Feb 3;11(3):e202200010. doi: 10.1002/open.202200010 (PMC8889503; doi:10.1002/open.202200010)
Supplement: Supplementary file 1 — Supporting Information [file OPEN-11-e202200010-s001.pdf]

ChemistryOpen

Supporting Information

Facet-Control versus Co-Catalyst-Control in Photocatalytic H_2 Evolution from Anatase TiO_2 Nanocrystals

Shanshan Qin, Lancang Shui, Benedict Osuagwu, Nikita Denisov, Alexander B. Tesler, and Patrik Schmuki*

Supporting Information

1. HR-SEM images of TiO_2 Oct obtained under various hydrothermal process conditions.

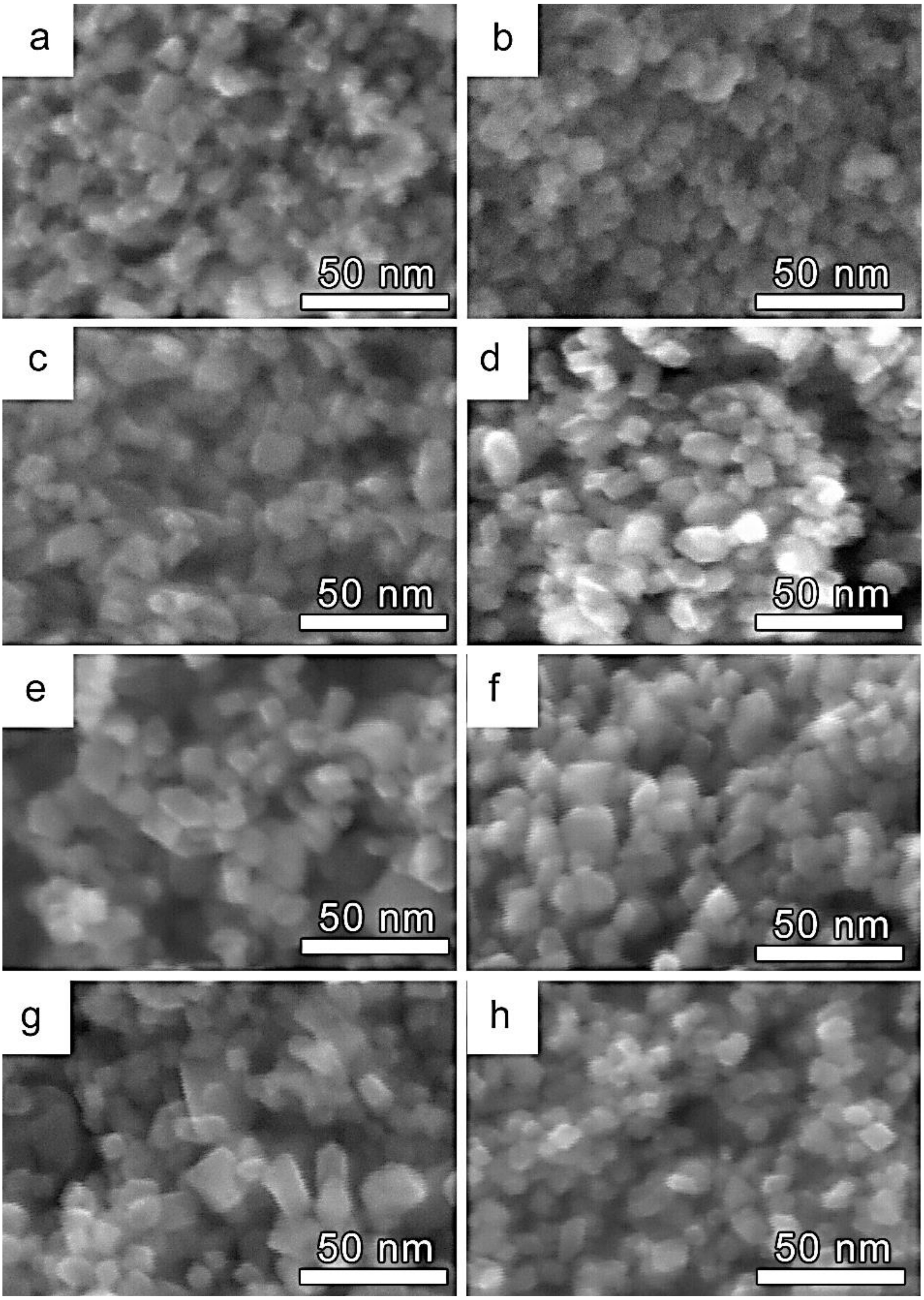

Figure S1. HR-SEM images of TiO_2 Oct obtained under various hydrothermal process conditions according to **Table S2**: (**a**) R7, (**b**) R8, (**c**) R9, (**d**) R10, (**e**) R11, (**f**) R12, (**g**) R13, and (**h**) R14.

2. HR-SEM images and corresponding EDX spectra of the as-formed and washed in NaOH TiO_2 NS.

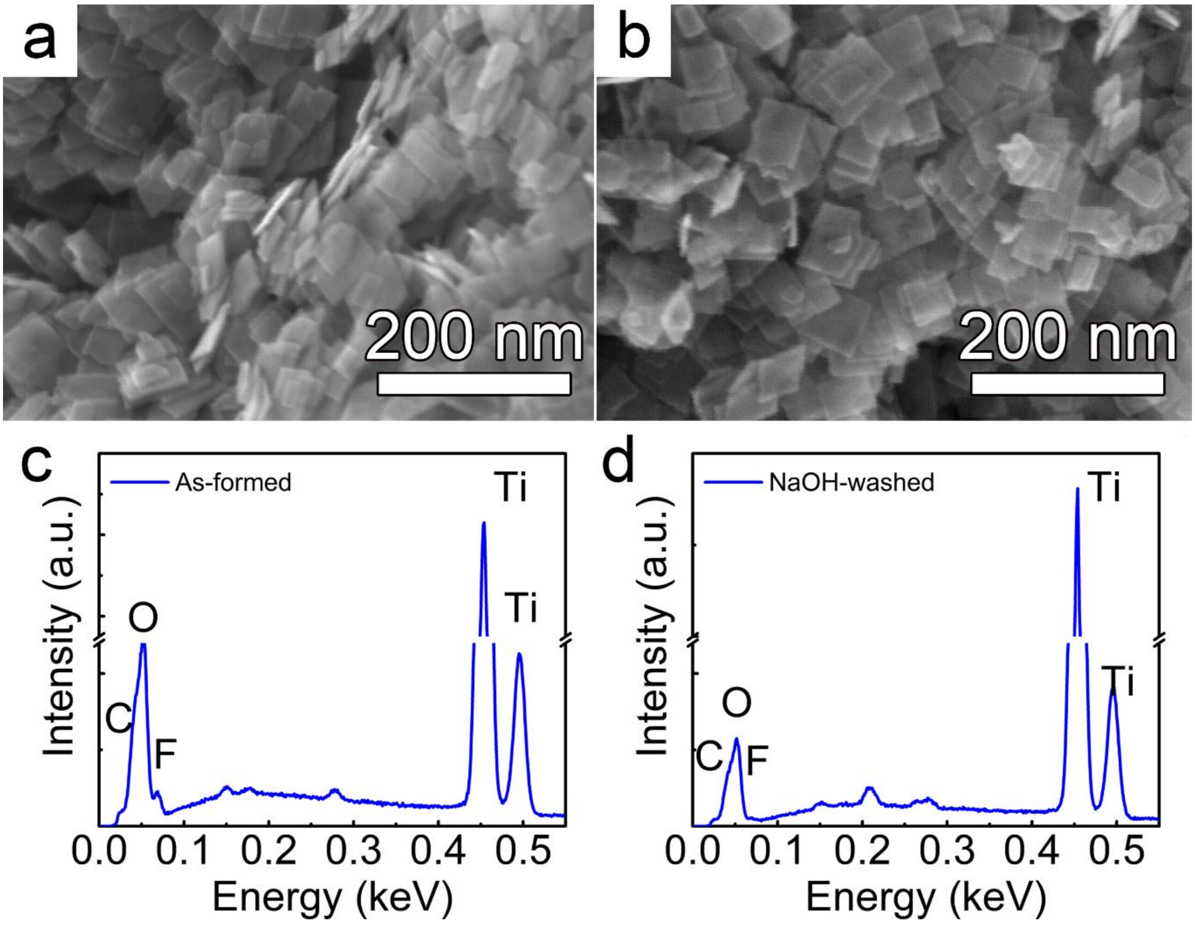

Figure S2. HR-SEM images and corresponding EDX spectra of the as-formed (**a**, **c**) and NaOH-washed (**b**, **d**) TiO_2 NSs.

3. High-resolution XPS spectra of the NaOH-washed TiO_2 NSs and the as-formed TiO_2 Oct.

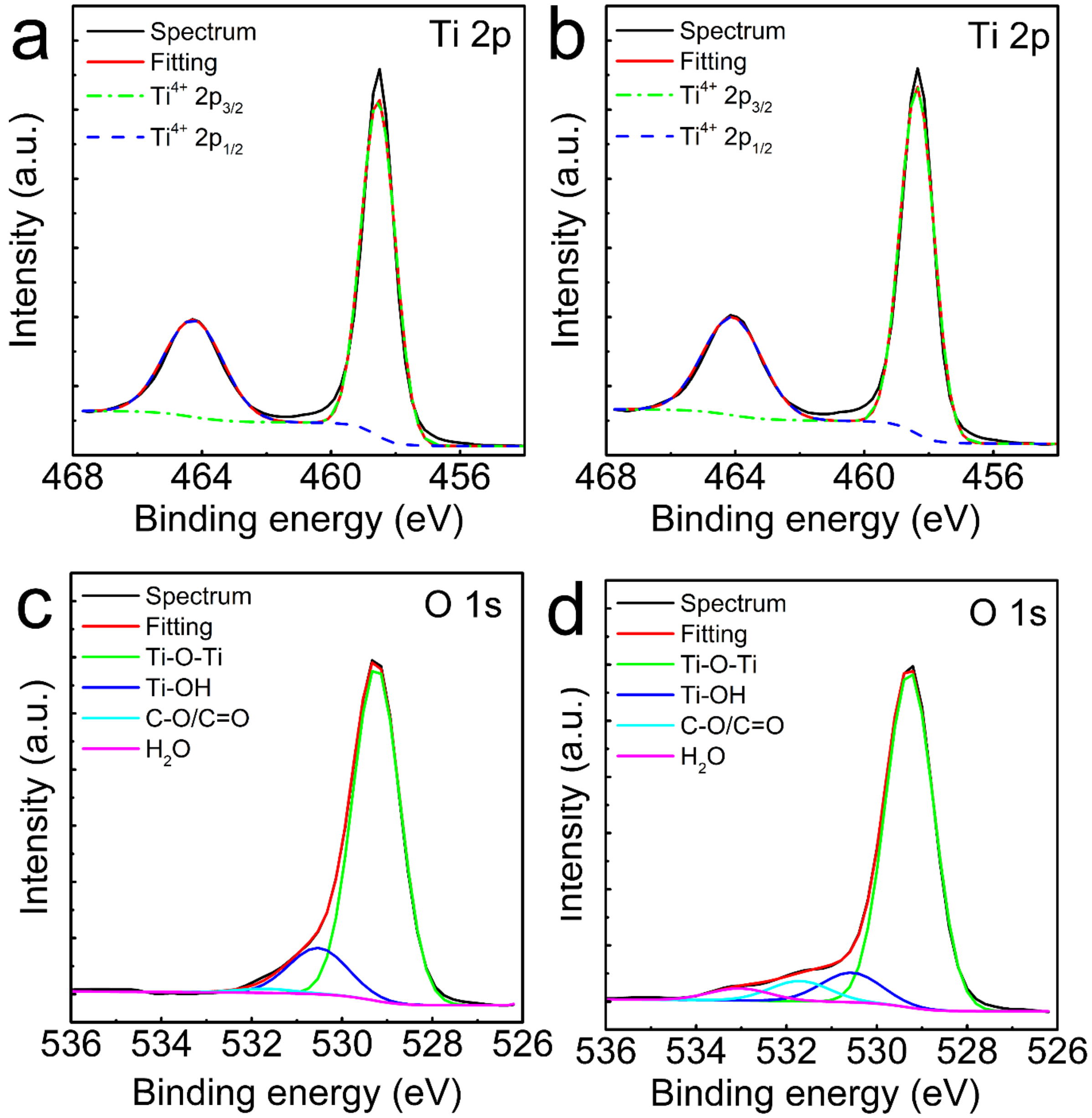

Figure S3. High-resolution XPS spectra of the NaOH-washed TiO_2 NSs and the as-formed TiO_2 Oct. (**a**) Ti 2p of TiO_2 NSs, (**b**) Ti 2p of TiO_2 Oct, (**c**) O 1s of TiO_2 NSs, and (**d**) O 1s of TiO_2 Oct.

4. Different reaction conditions of the $Ti(OH)_4$ precursor synthesis.

Table S1. Different reaction conditions were used for the synthesis of $Ti(OH)_4$ precursor.

	**V_{TiCl4} (mL)**	**V_{HCl} (mL)**	**$V_{5.5\ wt.\%\ NH3\cdot H2O}$ (mL)**	**$V_{4\ wt.\%\ NH3\cdot H2O}$ (mL)**
**Reaction 1**	6.6	30	30	38
**Reaction 2**	6.6	20	50	43
**Reaction 3**	6.6	100	70	32
**Reaction 4**	6.6	100	70	28
**Reaction 5**	6.6	100	70	30
**Reaction 6**	6.6	100	70	29

5. Different reaction conditions of the TiO_2 Oct synthesis.

Table S2. Different reaction conditions of the hydrothermal synthesis of octahedral anatase TiO_2 Oct.

Reaction	Precursor	$m_{precursor}$ (g)	Temperature (C)	Time (h)	Cooling
**Reaction 1**	1	2.0	180	24	Gradually
**Reaction 2**	2	2.0	180	24	Gradually
**Reaction 3**	3	1.0	180	24	Gradually
**Reaction 4**	4	1.0	200	24	Gradually
**Reaction 5**	4	1.0	160	24	Gradually
**Reaction 6**	4	1.0	180	18	Gradually
**Reaction 7**	4	1.0	180	27	Rapidly
**Reaction 8**	5	1.5	180	24	Rapidly
**Reaction 9**	5	1.0	180	35	Rapidly
**Reaction 10**	5	1.0	230	24	Rapidly
**Reaction 11**	5	1.5	230	24	Rapidly
**Reaction 12**	6	1.5	230	32	Rapidly
**Reaction 13**	6	1.0	230	40	Rapidly
**Reaction 14**	6	0.7	180	24	Rapidly
